# Immunological non-response and low hemoglobin levels are predictors of incident tuberculosis among HIV-infected individuals on Truvada-based therapy in Botswana

**DOI:** 10.1371/journal.pone.0192030

**Published:** 2018-01-31

**Authors:** Lucy Mupfumi, Sikhulile Moyo, Kesaobaka Molebatsi, Prisca K. Thami, Motswedi Anderson, Tuelo Mogashoa, Thato Iketleng, Joseph Makhema, Ric Marlink, Ishmael Kasvosve, Max Essex, Rosemary M. Musonda, Simani Gaseitsiwe

**Affiliations:** 1 Botswana Harvard AIDS Institute Partnership, Gaborone, Botswana; 2 Department of Medical Laboratory Sciences, School of Allied Health Professionals, University of Botswana, Gaborone, Botswana; 3 Department of Immunology and Infectious Diseases, Harvard TH Chan School of Public Health, Boston, Massachusetts, United States of America; 4 Department of Statistics, University of Botswana, Gaborone, Botswana; 5 Department of Biological Sciences, University of Botswana, Gaborone, Botswana; 6 College of Health Sciences, University of KwaZulu-Natal, Durban, Republic of South Africa; 7 Rutgers Global Health Institute, Robert Wood Johnson Medical School, Rutgers University, New Brunswick, New Jersey, United States of America; Public Health England, UNITED KINGDOM

## Abstract

**Background:**

There is a high burden of tuberculosis (TB) in HIV antiretroviral programmes in Africa. However, few studies have looked at predictors of incident TB while on Truvada-based combination antiretroviral therapy (cART) regimens.

**Methods:**

We estimated TB incidence among individuals enrolled into an observational cohort evaluating the efficacy and tolerability of Truvada-based cART in Gaborone, Botswana between 2008 and 2011. We used Cox proportional hazards regressions to determine predictors of incident TB.

**Results:**

Of 300 participants enrolled, 45 (15%) had a diagnosis of TB at baseline. During 428 person-years (py) of follow-up, the incidence rate of TB was 3.04/100py (95% CI, 1.69–5.06), with 60% of the cases occurring within 3 months of ART initiation. Incident cases had low baseline CD4+ T cell counts (153cells/mm^3^ [Q1, Q3: 82, 242]; p = 0.69) and hemoglobin levels (9.2g/dl [Q1, Q3: 8.5,10.1]; p<0.01). In univariate analysis, low BMI (HR = 0.73; 95% CI 0.58–0.91; p = 0.01) and hemoglobin levels <8 g/dl (HR = 10.84; 95%CI: 2.99–40.06; p<0.01) were risk factors for TB. Time to incident TB diagnosis was significantly reduced in patients with poor immunological recovery (p = 0.04). There was no association between baseline viral load and risk of TB (HR = 1.75; 95%CI: 0.70–4.37).

**Conclusion:**

Low hemoglobin levels prior to initiation of ART are significant predictors of incident tuberculosis. Therefore, there is potential utility of iron biomarkers to identify patients at risk of TB prior to initiation on ART. Furthermore, additional strategies are required for patients with poor immunological recovery to reduce excess risk of TB while on ART.

## Introduction

Tuberculosis (TB) is the leading cause of mortality by an infectious disease, ranking above HIV in current estimates [1]. In 2015, an estimated 1.4 million TB-related deaths occurred, a third of these among HIV infected individuals. In sub-Saharan Africa, a region that bears the brunt of these twin epidemics, 11% of incident TB cases occur among HIV-infected people [2, 3]. Strategies recommended by the WHO to reduce the TB burden include intensified case finding, isoniazid preventative therapy, and early ART initiation for TB-HIV co-infected patients.

The survival benefits of initiation of combination antiretroviral therapy (cART) in co-infected patients, particularly those with low CD4+ T-cell counts, have been well documented [4, 5]. However, although cART has been reported to reduce the risk of developing active TB among HIV infected persons by 67% [6], co-infected individuals have a heightened risk of mortality [7, 8]. Furthermore, HIV infected individuals on cART remain at a higher risk of developing active TB than HIV uninfected individuals [9–11]. Studies conducted in both low and high income countries have determined that the risk of active TB does not return to background rates despite long term ART [2, 3, 11–13].

The mechanisms driving the heightened risk of TB among HIV infected individuals are not well understood. The most recognized immune defect caused by HIV is the absolute reduction in CD4+ T-cells, the mainstay of the immune response to TB [14, 15]. This accounts for the increased risk of TB in HIV-infected persons, which is strongly associated with the progressive loss of CD4+ T-cells [16]. However, the fact that the risk of TB remains high even in early HIV infection or with immune restoration while on cART shows that HIV infection confers qualitative changes to the functionality of the CD4+ T-cells and other aspects of the immune response to TB [17, 18].

Other well recognized factors accounting for this elevated risk include a low hemoglobin, low BMI, and increasing age [16, 19–22]. Low BMI is a strong independent predictor of mortality among HIV infected individuals even in the context of ART initiation [23], with higher BMI levels associated with a protective effect on both mortality and incident TB estimates [23, 24]. HIV-infected individuals are therefore likely to benefit from micronutrient supplementation, although there have been conflicting reports of their effect on HIV-related co-morbidities or mortality [25]. Similarly, determinants for TB among HIV-infected patients vary across settings [26].

Understanding the context-specific incidence and predictors of TB during cART remain important keys to designing effective interventions to mitigate the ravenous effects of the syndemic. Furthermore, most studies describing burden of TB in ART programs have been in patients on zidovudine-containing regimens, there has only been one study conducted in patients on a Tenofovir-based ART regimen [11]. In Botswana, currently listed as an HIV-TB high burden country[1], high mortality rates have been reported among co-infected patients despite provision of ART and anti-tuberculosis therapy (ATT) [27]. It is therefore critical to determine factors associated with risk of incident TB to allow early identification of patients that will benefit from isoniazid preventative therapy. We conducted a retrospective analysis of data collected during an observational study evaluating efficacy of a Truvada-based cART backbone in Botswana to determine the TB incidence and the associated predictive factors.

## Materials and methods

### Study design and participants

We conducted a retrospective analysis of data from 300 adults enrolled into an observational study, evaluating the efficacy and tolerability of a Truvada-based regimen in HIV-1C infected adults—“*Bomolemo study*”—conducted in Gaborone between November 2008 and July 2011[28]. In 2008, Botswana adopted Tenofovir plus Emtricitabine (Truvada^™^) combined with either Efavirenz or Nevirapine as its first line ART regimen. The “*Bomolemo study”* was designed to demonstrate the tolerability, virologic and immunologic response of a Truvada-containing regimen for HIV-1 C infected adults in Botswana. Participants were HIV infected, ART-naïve and aged 18 years and older. Additional eligibility criteria included the presence of an AIDS defining condition or a CD4+ T-cell count <250cells/mm^3^, consistent with WHO guidelines at the time. Female participants were asked to provide a urine specimen for pregnancy testing and were excluded if they were pregnant or had received single dose Nevirapine as PMTCT within the 6 months preceding enrollment. After study entry and ART initiation, participants were scheduled for evaluations at 1 month and then every 3 months until the final study visit at week 96. At these visits, physical examination and medical history was taken in addition to laboratory tests for CD4, viral load, hematology, and chemistry. This study received ethical approval from the Botswana Ministry of Health’s Health Research Development Committee (PPME-13/18/1) and the Harvard T.H. Chan School of Public Health IRB (16470–02). All participants provided a written informed consent including use of their collected data.

### Laboratory assays

HIV and chronic hepatitis B viral (HBV) infection was diagnosed by double ELISA and surface antigen testing respectively. Viral load samples were processed on the Roche Amplicor HIV-1 monitor and CD4 T-cell measurements on the BD FACSCalibur at the Botswana Harvard HIV reference Laboratory (BHHRL). For this analysis, we defined immunologic non-response as CD4+ T-cell count gains of less than 20% within 6 months of cART initiation [29] and virologic suppression as viral load less than 400copies/ml after 6months on cART.

### Tuberculosis screening and diagnoses

Tuberculosis was diagnosed and treated through the national programme per the Botswana National TB treatment guidelines. Briefly, tuberculosis diagnosis was based on either a positive sputum AFB or culture result, or abnormal chest radiology. Patients on TB treatment with neither abnormal chest x-ray nor microbiological confirmation were classified as “clinical TB”. Information on clinical diagnoses was collected at each study visit and documented on a case report form. For this analysis, any patient reported as having tuberculosis with a documented diagnosis date was recorded as a case. We also defined prevalent TB cases as those who had a TB diagnosis at baseline and incident TB cases as those diagnosed with TB after cART initiation.

### Data analysis

Person-time at risk was accrued from the date of cART initiation until death or study closeout or, in the case of those who developed incident TB, date of first recorded TB diagnosis. Incident TB rate was calculated as the number of new cases of TB divided by total person-years (PY) at risk and expressed per 100py. Kaplan-Meier estimator of the survivor function was used to generate a TB-free survival curve and to determine the impact of poor immunological response on TB incidence, adjusting for gender. Univariate and multivariate Cox proportional hazard models were generated adjusting for CD4, gender and age to evaluate risk factors for incident TB during ART. Variables with p-values < 0.05 were included in the multivariate model. All statistical analyses were conducted in Stata version 14.2 (College Station, Texas, USA).

## Results

### Cohort characteristics

The median age of the cohort was 36 years (Q1, Q3: 32, 42), and 64% were females [Table 1]. Overall, participants had advanced immunodeficiency, with median CD4 counts of 168 cells/mm^3^ (Q1, Q3; 86, 232) and log HIV viral load of 5.1cps/ml (Q1, Q3: 4.6, 5.6). Participants with prevalent TB had lower hemoglobin levels (11.2g/dl; Q1, Q3: 8.9, 13), although this was not significantly different from the rest of the participants.

**Table 1:** Characteristics of cohort at enrolment.

*Variable*	*Characteristic*	*All patients*	*Prevalent TB*	*Non-TB*	*p-value*
	N	300	45	255	
*Demographics*	Gender				
	female	192 (64%)	20 (10%)	172 (90%)	<0.01^1^
	male	108 (36%)	25 (23%)	83 (77%)	
	Age (years), Median (Q1,Q3)	36 (32, 42)	37 (33, 45)	36 (31, 42)	0.15
*BMI*	Median (Q1,Q3)	21.6 (19.1, 25.1)	19.8 (17.6, 23.3)	21.5 (19.1, 24.6)	<0.01
*Hemoglobin (g/dl)*	Median (Q1,Q3)	11.5 (9.9, 12.9)	11.2 (8.9, 13)	11.6 (10.1, 12.9)	0.28
CD4 cell count*	Median (Q1,Q3)	168 (86, 232)	119 (68, 224)	168 (92, 238)	0.05
	> 250cells/μl	237 (79%)	9 (15%)	51 (85%)	
	≤ 250cells/μl	60 (20%)	36 (15%)	201 (85%)	0.58
*Viral load (log) (cps/ml)*	Median (Q1,Q3)	5.13 (4.6, 5.6)	5.5 (4.9, 5.8)	5.1 (4.6, 5.5)	<0.01
*Hepatitis B*	Chronic HBV	28 (9%)	3 (7%)	25 (10%)	0.78^2^

*3 missing baseline CD4 counts;

^1^Chi-square test,

^2^Fisher’s exact, the rest Wilcoxon signed rank test

Q1-1^st^ quartile, Q2, 3^rd^ quartile, g/dl-grams per deciliter, cps/ml-copies per milliliter

### TB diagnosis

**Fig 1:**
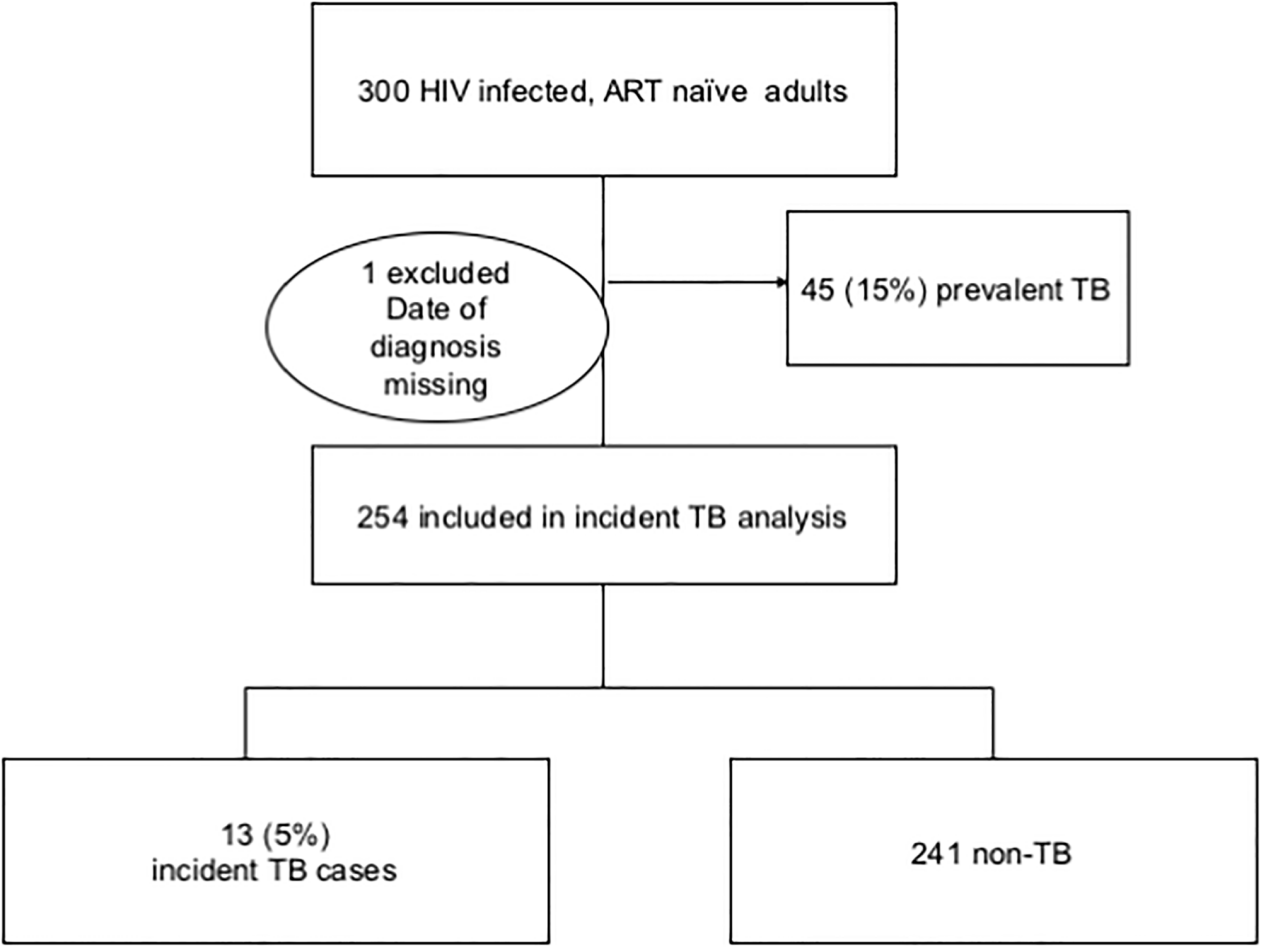
Participant flow diagram of individuals enrolled in the incident TB cohort analysis. Forty-six individuals were excluded from further analysis: 45 had TB at baseline (prevalent TB) and one did not have a recorded date of diagnosis.

Forty-five (15%) participants had TB at baseline (prevalent TB) and were more likely to be male (p<0.01), have lower BMI (p<0.01), and higher HIV viral loads (p<0.01) [Table 1]. These participants were excluded from further analysis [Fig 1]. The remaining participants contributed 428 person-years of follow-up. Incident TB was diagnosed in 13 of 254 [5%, IR 3.04/100py (95% CI, 1.69–5.06)] participants within a median of 41 days (Q1, Q3:28, 78) from enrolment. Bacteriological confirmation was available in 6 (46%) of the incident cases. The remaining cases were placed on treatment based on abnormal chest radiology (23%) or clinical indication (31%).

### Incident TB and CD4 counts

**Fig 2:**
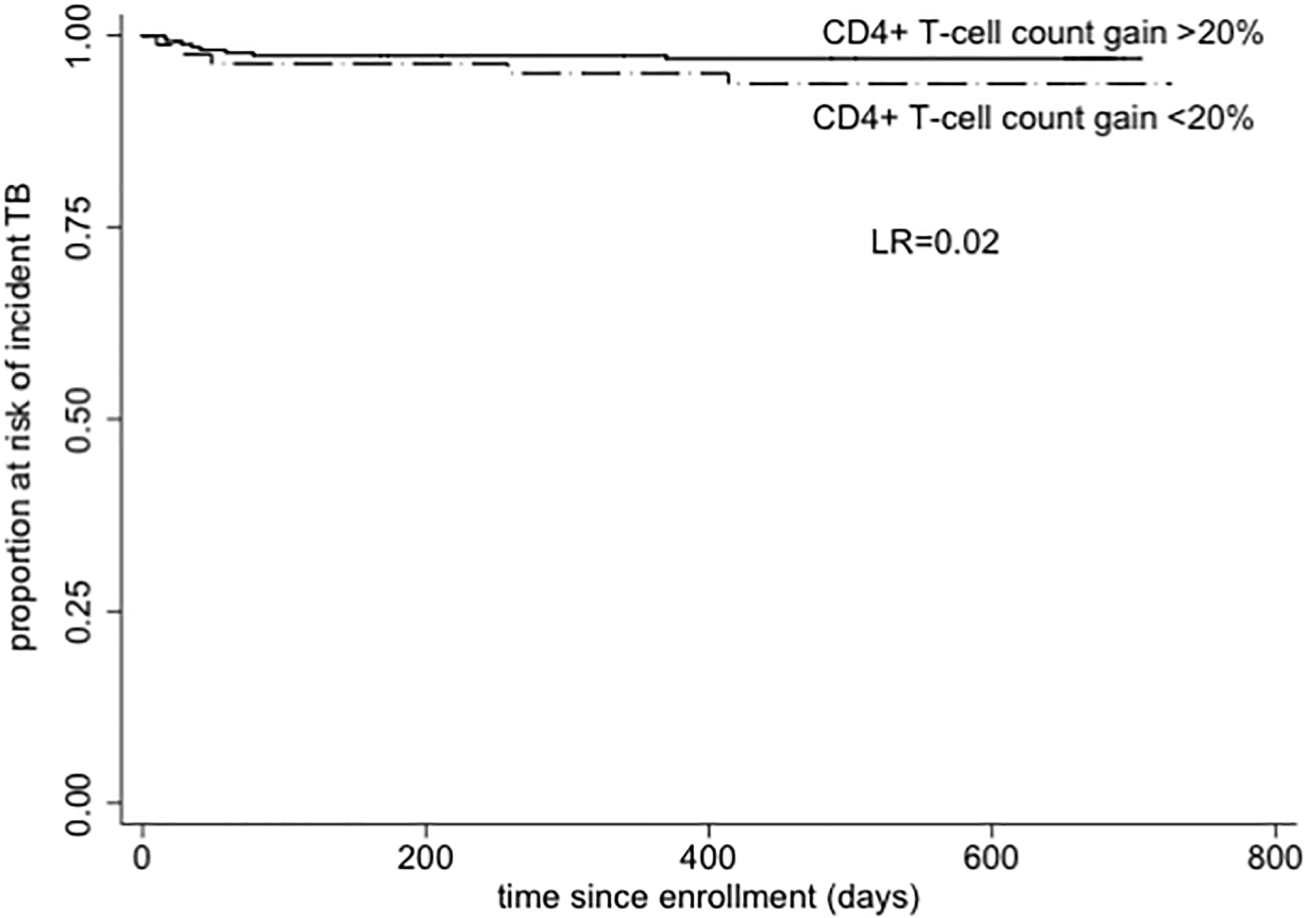
Kaplan Meier plot of time to incident TB in patients with poor immunological response. Participants who failed to achieve a CD4+T-cell count increase of at least 20% of the baseline counts by 6months of follow-up were classified as poor immunological responders.

Throughout the follow-up period, males had significantly higher CD4+T-cell counts than females (p<0.01), although there were proportionally more males with incident TB (Table 2). The median CD4+T-cell count for the incident TB cases was 153 (Q1, Q3: 82, 242) cells/ mm^3^ (Table 2). The risk of developing incident TB was not associated with immunologic non-response [HR 2.99 (95% CI 0.98–9.15), p = 0.06] (data not shown), however, immunologic non-responders had a shorter time to diagnosis [log rank p = 0.02, Fig 2].

**Table 2:** Baseline features associated with incident TB in patients on ART.

*Characteristic*	*Incident TB*	*Non-TB*	*p-value*
*N*	13	241	
*Female, n (%)*	6 (3)	166 (97)	0.08
*Male, n (%)*	7 (9)	75 (91)	
*Age in years* *(median, Q1, Q3)*	34 (32, 37)	36 (31, 42)	0.33
*BMI* *(median, Q1, Q3)*	18.1 (16.2, 19.8)	22.1 (19.4, 25.8)	<0.01
*Hemoglobin g/dl* *(median, Q1, Q3)*	9.2 (8.5, 10.1)	11.6 (10.3, 12.9)	<0.01
*CD4 cells/mm^3^* *(median, Q1, Q3)*	153 (82, 242)	170 (93, 235)	0.69
*CD4*>*250cells/μl, n (%)*	3 (6)	48 (94)	
*CD4*≤ *250 cells/μl, n (%)*	10 (5)	190 (95)	0.51
*Viral load (log) (cps/ml)* *(median, Q1, Q3)*	5.3 (5.1, 5.4)	5.0 (4.6, 5.5)	0.24
*Chronic HBV, n (%)*	3 (23)	22 (9)	0.12

### Risk factors for incident TB

Incident cases had significantly lower baseline BMI and hemoglobin levels compared to non-TB cases (Table 2). In univariate analysis, the risk of incident TB was higher among participants with low BMI [HR 0.73 (95% CI 0.58–0.91) p<0.01] and low baseline hemoglobin levels [≤8g/dl; HR 10.84 (95% CI 2.93–40.06) p<0.01] (Table 3). In additional analysis, participants with CD4+T-cell counts below 250cells/mm^3^ by one year of follow-up were at significant risk of incident TB [HR 5.48 (95% CI 1.66–18.2) p<0.01 (results not shown)].

**Table 3:** Multivariable risk factor analysis for incident TB among patients on ART.

	*Unadjusted*			*Adjusted*		
*Characteristic*	HR	95% CI	p-value	aHR	95% CI	p-value
*Male*	2.45	0.82–7.30	0.11	-	-	-
*Age in years*	0.97	0.90–1.04	0.34	-	-	-
*BMI*	0.73	0.58–0.91	0.01	0.81	0.66–1.00	0.05
*Hemoglobin (<8g/dl)*	10.84	2.99–40.06	<0.01	6.88	1.78–26.58	0.01
*Baseline CD4 count*	1.00	0.99–1.00	0.55	-	-	-
*Baseline viral load (log)*	1.75	0.70–4.37	0.23	-	-	-
*Hepatitis B infection*	2.85	0.78–10.35	0.11	-	-	-

In the multivariate model, only baseline hemoglobin levels were significantly associated with incident TB [adjusted HR 6.88 (95% CI: 1.78–26.58) p = 0.01; Table 3]. We also found a significant interaction between gender and BMI [adjusted HR 0.61 (95% CI: 0.38–0.96) p = 0.03] (data not shown). However, there was no interaction between gender and hemoglobin level [adjusted HR 1.08 (95% CI 0.96–1.22) p = 0.19] (data not shown).

## Discussion

We have shown in this study a TB incidence rate of 3.04/100py over a 2-year follow-up period of patients on a Truvada-based cART regimen. As is the case with ART programs in sub-Saharan Africa, 70% of the incident TB cases occurred within the first three months of cART initiation. Additionally, there was a significant association between anemia and incident TB, highlighting the need to identify biomarkers that will allow timing of appropriate interventions. Patients with poor immunologic recovery by one year on cART and those with low CD4 counts had an increased risk of and shorter time to incident TB disease.

The reduction in TB risk attributable to cART varies depending on the background risk of transmission [30]. Studies have shown that cART reduces the individual risk of TB disease among HIV-infected persons by between 60 and 90% irrespective of CD4 count [20, 31, 32]. However, this reduction is time dependent, decreasing from 3.6/100py in the first year to 1.0/100py by the fifth year on cART [20]. Furthermore, by year three on treatment, patients still have a 5–10 times higher risk of TB compared to HIV-uninfected [31, 33]. A retrospective analysis of data from Wales confirmed these findings, concluding that the risk of TB in HIV infected patients does not reach background rates in black Africans [12]. This is likely due to the fact that long-term restoration of immune phenotype and function is limited despite a long duration on cART [34], particularly in patients with low CD4 counts.

In this study, patients with advanced immunodeficiency were recruited, reflecting the cART eligibility guidelines of the Botswana HIV Treatment Programme at the time. The risk of development of active TB is associated with lower nadir CD4 counts and those with suboptimal immunological responses have an increased risk of developing active TB. As we demonstrated, it is therefore time-updated and not baseline CD4, that is the strongest predictor of incident TB. In our study, patients who failed to achieve a 20% gain in baseline CD4 counts by 6 months on cART had a 2-fold higher risk of TB. Other studies have reported that patients with the lowest baseline CD4 counts remain at increased risk of TB for a longer period of time compared to those with high CD4 counts [35]. Furthermore, updated CD4 counts closer to the time of diagnosis have also been reported to be stronger predictors of incident TB than baseline CD4 counts [11].

Immunological non-recovery is a known predictor of the immune reconstitution syndrome (IRIS) that complicates management of patients on cART [35–38]. Patients who fail to achieve at least a 20% increase in CD4 counts on cART are considered to be immunological non-responders [29]. The reasons for this non-response in the context of viral suppression are not well understood but may be linked to a lower CD4 nadir [29] or the elevated levels of T-regulatory cells in circulating blood [39]. Previous work in our setting showed that immunological non-recovery in TB/HIV co-infected patients was a significant risk factor for TB-IRIS and early mortality and this was associated with elevated levels of the pro-inflammatory cytokine, IL-6 [40]. Most of the patients analyzed in this study had low CD4 counts prior to cART initiation and this may account for the high incidence rate reported in this study, although our estimate is consistent with what has been reported in similar high burden settings [32, 33]. In contrast, studies from other African countries have reported lower incidence rates in patients on cART [11, 41, 42]. This could possibly be a reflection of the lower background rates of prevalent TB or inadequate screening or documentation of TB cases in these settings [41].

Consistent with reports from similar high burden settings [3, 16, 20, 43, 44], more than half of the incident cases occurred within 6 months of cART initiation. This possibly demonstrates the difficulty in screening patients for TB and highlights the need for biomarkers that can identify patients at increased risk of TB at the time of cART initiation. The TB symptom screen algorithm proposed by Cain and colleagues [45] may be less sensitive and specific among individuals taking cART [16]. Cases were identified within a short period of cART initiation, thus likely represent subclinical disease [16, 20, 44] or TB-IRIS [20]. The fact that this finding has also been demonstrated in a study that recruited patients with CD4 counts >350cells/μl [16] shows that enhanced diagnostics and intensified case finding, in addition to early cART initiation, will be required to curb the challenge of incident TB in ART programs in Africa.

An interesting finding from this study is the heightened risk of incident TB in patients with severe anemia, defined as a hemoglobin level less than 8g/dl [46]. Anemia is a common comorbidity reported at TB diagnosis in 32–86% of patients [22]. Patients in our study were on a Tenofovir-based regimen, which does not induce anemia as is the case with zidovudine. This suggests there is another mechanism driving the intricate relationship between anemia and TB. It has been postulated that severe anemia in patients with undiagnosed TB is likely due to the pro-inflammatory cytokine activation that induces an anemia of chronic disease [22, 47]. IL-6 upregulates the transcription of hepcidin, the iron regulatory protein, which leads to reduction in iron absorption and sequestration of iron by macrophages and enterocytes [48], limiting iron delivery to erythroblasts thus causing a functional anemia. This increased iron storage in macrophages promotes replication of bacilli thus markers of iron homeostasis may be early markers of risk of progression from latency to active TB. Studies have also shown that time-updated anemia severity is an independent predictor of mortality in patients on ART [11, 22], which may suggest a role for iron biomarkers in screening strategies for TB prior to cART initiation. Iron biomarkers also have the potential to contribute to the development of risk profiles for the progression from latent TB infection to active disease [22], a current area of active research.

Nutritional status as measured by BMI, was an independent predictor of incident TB in this cohort, in concordance with reported studies from the region [11, 20, 21, 43]. There is a bidirectional relationship between weight loss and TB. People with TB most often have a loss of appetite that results in weight loss, a main symptom of TB disease [49]. Individuals with increased BMI may have higher daily protein and energy intake which could result in a more robust immune function and drive the reduction in both incident TB and mortality [23]. BMI category change has also been reported as a marker of favorable treatment outcomes [24].

HIV induces early aging of the immune system, which likely contributes to reactivation of latent TB and therefore heightened risk of incident TB in older ages [30]. Other studies have also reported an association between gender and incident TB [20, 50], although we did not observe such an effect. We observed significant interaction between gender and BMI, which possibly masked the true effect of BMI in our multivariate model. This is consistent with findings from a large cohort study of patients on ART in Nigeria that showed that gender is an effect modifier of the association between BMI and anemia with TB, suggesting that males may benefit from nutritional interventions to boost their immunity to TB [11].

Incident TB may have been overestimated due to most cases appearing within the first 6 months of ART. Furthermore, some cases may have died before a TB diagnosis which may bias our estimates as we did not perform a competing risk analysis. However, our findings are similar to estimates reported from similar high-burden settings[10, 20, 43]. The combination of both a high rate of prevalent and incident TB in this study highlights the magnitude of the HIV-associated TB epidemic in ART programs in low-resource setting. While ART initiation at high CD4 counts is expected to reduce incident TB rates[16, 20, 35], there is need to strengthen TB screening algorithms and combine with biomarkers to ensure that patients at increased risk of TB are identified and appropriate interventions instituted.

The combination of cART and isoniazid preventive therapy would be useful in reducing the burden of TB in ART programs in Africa[51]. However, the risk of infection post-IPT remains considerably higher in high incidence compared to low incidence settings [52] even when IPT is extended to 36 months [53]. While it is not clear if these incident cases post-IPT represent a failure to sterilize latent infection at the time of IPT initiation or reinfection, it is generally accepted that these are a function of TB endemicity [53]. Nonetheless, there is no disputing the survival benefit of combined IPT and ART in high incidence settings. Multivariate algorithms that rely on accurate classification of latent infection and better treatment regimens for IPT are therefore urgently needed[54].

Our results should be interpreted in light of the strength and weaknesses of the study design. The main weakness of the study is the retrospective study design that has inherent weaknesses. Tuberculosis was not actively investigated in the main study and relied on clinical investigation as per the standard of care through the routine health system. Consequently, over half the incident cases had no laboratory confirmation, which may have introduced misclassification bias and overestimation of our incident cases. However, most TB cases are diagnosed empirically in Africa even in settings provided with the GeneXpert test [44, 55]. Secondly, this study was conducted at a time when ART guidelines recommended initiation at CD4 count less than 250cells/mm^3^ so the findings of this analysis may not be generalizable to current guidelines. However, studies conducted in patients with CD4 count above 350cells/ mm^3^ suggest that incident TB is still a problem [16] thus pointing to the fact that multiple interventions will be required in order to mitigate the burden of HIV-associated tuberculosis.

In conclusion, we have reported a high rate of incident TB among patients on ART in Botswana and highlight a possible role for iron biomarkers in identifying patients at risk of TB. Longitudinal studies are warranted in the HIV test and treat era to define true estimates of incident TB and determine the predictive value of iron biomarkers.
