# Correction: Immunological non-response and low hemoglobin levels are predictors of incident tuberculosis among HIV-infected individuals on Truvada-based therapy in Botswana

**DOI:** 10.1371/journal.pone.0198711

**Published:** 2018-06-01

**Authors:** Lucy Mupfumi, Sikhulile Moyo, Kesaobaka Molebatsi, Prisca K. Thami, Motswedi Anderson, Tuelo Mogashoa, Thato Iketleng, Joseph Makhema, Richard Marlink, Ishmael Kasvosve, Max Essex, Rosemary M. Musonda, Simani Gaseitsiwe

The ninth author's name is spelled incorrectly. The correct name is: Richard Marlink.
